# The Autonomic Signature of Guilt in Children: A Thermal Infrared Imaging Study

**DOI:** 10.1371/journal.pone.0079440

**Published:** 2013-11-19

**Authors:** Stephanos Ioannou, Sjoerd Ebisch, Tiziana Aureli, Daniela Bafunno, Helene Alexi Ioannides, Daniela Cardone, Barbara Manini, Gian Luca Romani, Vittorio Gallese, Arcangelo Merla

**Affiliations:** 1 Department of Neuroscience, Section of Human Physiology, Parma University, Parma, Italy; 2 Institute of Advanced Biomedical Technologies (ITAB), G. d'Annunzio Foundation, Chieti, Italy; 3 Department of Neuroscience and Imaging, University of Chieti-Pescara, Italy; St. Joseph's Hospital and Medical Center, United States of America

## Abstract

So far inferences on early moral development and higher order self conscious emotions have mostly been based on behavioral data. Emotions though, as far as arguments support, are multidimensional notions. Not only do they involve behavioral actions upon perception of an event, but they also carry autonomic physiological markers. The current study aimed to examine and characterise physiological signs that underlie self-conscious emotions in early childhood, while grounding them on behavioral analyses. For this purpose, the “mishap paradigm” was used as the most reliable method for evoking feelings of “guilt” in children and autonomic facial temperature variation were detected by functional Infrared Imaging (fIRI). Fifteen children (age: 39–42 months) participated in the study. They were asked to play with a toy, falsely informed that it was the experimenter's “favourite”, while being unaware that it was pre-planned to break. Mishap of the toy during engagement caused sympathetic arousal as shown by peripheral nasal vasoconstriction leading to a marked temperature drop, compared to baseline. Soothing after the mishap phase induced an increase in nose temperature, associated with parasympathetic activity suggesting that the child's distress was neutralized, or even overcompensated. Behavioral analyses reported signs of distress evoked by the paradigm, backing up the thermal observation. The results suggest that the integration of physiological elements should be crucial in research concerning socio-emotional development. fIRI is a non invasive and non contact method providing a powerful tool for inferring early moral emotional signs based on physiological observations of peripheral vasoconstriction, while preserving an ecological and natural context.

## Introduction

Moral development is a process that emerges early on in human life and unfolds gradually through the interplay of the conscious self and the feedback received by social surroundings. As an important part of that development, the accompanying emotions provide children with the motivational force to attain a higher level of “fitness” between the self and the others, helping them to appreciate the link between the moral standards of their social community and their own moral behavior. Moral emotions are thought to function like a “barometer” [1], which gives the children an immediate and salient measure on the moral acceptability of their actions, whether they signal a transgression or an accomplishment of the social norms, such as shame and guilt, in the former case or pride and sympathy in the latter [2]]–[[4].

Given their key role in the socialization process, the vast majority of research focused more on negative than positive emotions. Since guilt and shame are thought to help children prevent aversive conducts [5]–[6], they are both considered “quintessential moral emotions”, compared to other emotions of the same family [7] (p. 447); and, between the two, guilt is judged more moral than shame [8]. Since it arises from the awareness of a wrongdoing, as opposed to the self as a bad entity [7], guilt motivates individuals to take care of other's feelings, thus leading them to respond to the transgression by restitution and reparation rather than by avoidance and hiding. Monitoring the development of guilt from an early age could therefore be compelling in providing a reliable mosaic of child moral development at the very outset [8]]–[[11].

Classical theories grounded the guilt experience in the mechanism of moral internalization, focusing on early school years as critical periods for moral development [12]]–[[14]. Evidence [5], [15], has indicated such that children's concern about their own wrongdoings, as well as their proneness to act appropriately for reparation, occurs even in toddler period. Even researchers reluctant to identify guilt as a distinct emotion at an early age [4], acknowledged the above evidence, as a signalling the earliest steps of guilt development. In extent, according to a functionalist point of view positing that emotions have an adaptive function and are defined not only by cognitive requisites, but also by the functions they serve in relevant contexts. Agreement now exists that discomfort showed by younger children, after causing or after thinking to have caused damage to another person can be considered as a reliable antecedent of future guilt [4]. Based on that consensus, the experience of guilt is assigned to younger children, if they show a coherent pattern of guilt-relevant behaviours in guilt-relevant situations: children must provide evidence of distress at expressive, postural and vocal level as well as of reparative behaviours at an action level, and that evidence must be provided just after a transgression happened.

To have both requisites fulfilled, past studies used a task, involving an appropriate transgression (“mishaps”) in naturalistic yet controlled conditions. Coherent results have been obtained by a number of studies in the same direction [3]]–[[5], [16], [17].The mishap paradigm is considered a reliable situation for experimentally eliciting such a “blend of affective and behavioral signs of discomfort” [4], (p. 462), thought to reflect future guilt.

Whereas the behavioral signs of early guilt have been identified, physiological signs have largely been ignored. However, bio-behavioral signs of higher order moral emotions should be considered. Many recent theories of emotion [18] view the autonomic nervous system (ANS) as a major component of the emotional response. The “somatic marker hypotheses” advances that somatic arousal is the connecting link between previous events and the feelings associated with the representations of those events [19]. On a neural level, the ventromedial prefrontal cortex, which is described as the focal area for guilt processing, regulates and evaluates the emotional value of sensory stimuli further projecting to the basal forebrain, and the brainstem regions, enabling the activation of physiological bodily components of an emotional response [20]. Thus, the ANS might not only be the derivative of an emotional response, but rather a window for observing neo-cortical and sub-cortical healthy development, signifying normal moral acquisition.

Hence, it would be important to investigate ANS physiological responses in emotional research [18], [21], [22]. Only a couple of studies, to our knowledge, focused on autonomic correlates of moral emotions in early childhood. In the most recent one [23], in a sample of children ranging from 3.7 to 4.5 years of age, positive associations were observed between cortisol levels and shame proneness, confirming a previous study evidence [24] that found a higher cortisol response being associated with a greater expression of shame. However, since saliva samples were collected in that study at six different time points to obtain cortisol responses, a quite interfering procedure was adopted not allowing contact free physiological measures.

The current study aims to contribute to research, on the physiological nature of early moral emotion by focusing on guilt. We sought to detect the autonomic signs of sympathetic arousal in children aged 39–42 months by means of online monitoring of ANS activity during the mishap paradigm [3], [17]. For this purpose and in order to preserve the naturalistic, however controlled, situation provided by that paradigm, we employed the functional infrared imaging (fIRI) technique [25]. fIRI is a contact-free and non-invasive methodology, which estimates variations on autonomic activity reflected by cutaneous temperature modulations by means of recording the thermal infrared signals released by the human body [25]]–[[29]. The reliability of fIRI has been proven with the use of simultaneous recordings, grounding fIRI on GSR, confirming that fIRI provides adequate detection power for physiological recordings [26]. fIRI provides a reliable tool that enables one not only to infer psycho-physiological excitement, but also differentiate between baseline and affective states [17], [31]. In particular, during emotional or physical threat a complex ‘play’ of cutaneous heat variation takes place, involving skin tissue, inner tissue, local vasculature, and metabolic activity. The above ‘acting’ mechanisms controlled by the autonomic nervous system are the driving force of a physiological functional thermal observation. Because of its characteristics, fIRI is particularly useful for observing emotions in infancy research, when the individuals cannot express their feelings verbally and are difficult to engage in strictly controlled experimental settings [32]. In general, it represents a very advantageous opportunity for the developmental field as a whole, since children can be left free to exhibit their spontaneous behavior when adjusting to the experimental setting.

Innervations from both the sympathetic nervous system (SNS) and the parasympathetic nervous system (PNS) are commonly received by all body tissue. During threat the SNS causes sweat secretions that lubricate the skin, achieving elasticity [33], [34], as well as sustaining temperature homeostasis in prolonged periods of vigorous activity [35]]–[[38]. Furthermore, vasoconstrictions of the blood vessels of the skin protect the body from possible hemorrhage and excessive blood loss during injury [39]]–[[42]. The above physiological occurrences, especially prevalent when emotional stimuli are present in the proximal environment [18], cause skin temperature to fluctuate as a derivative of SNS activity and subcutaneous vasoconstriction. Thus, by observing the thermal infrared signal, one can infer autonomic arousal and further differentiate between the two competing subdivision of the ANS.

The nose has been the most reliable region for detecting psycho-physiological arousal [17], [25], [31], [32], [43]]–[[48]. Therefore, in the present study temperature observations for the alternating autonomic states will be based on the nasal tip. . It is expected that children will show autonomic signs of distress associated with the behavioral signs of discomfort [4]. In particular, we expect a temperature drop at the nasal tip after the transgression due to subcutaneous vasoconstriction, whereas positive emotional states (playing/social interaction) are expected to have the opposite effect on nasal heat due to vasodilation and returning to autonomic equilibrium.

## Methods

### Ethics Statement

Full explanation of the procedure of the experiment was given to the parents of the children and all questions were answered verbally prior to the study. The parents provided their written consent and for the publication of the photograph of child 7, the guardian of the child has given written informed consent, as outlined in the PLOS consent form. Ethical approval for the study was given on January 27^th^ 2010 (prot. 37) by the Local Ethics Committee (Comitato di Etica per la Ricerca Biomedica, Universita Degli Studi G.D' Annunzio, Azienda Sanitaria Locale – Chieti) for the study of *Emotions and the development of intersubjectivity: A functional Infrared Imaging Study*. All procedures were in line with the Declaration of Helsinki.

### Participants

Participants included 20 children (8 females & 12 males; age range = 39–42 months, M = 40.38 months, SD = 0.87 months), of which 15 could be processed entirely and included in the final statistics (7 males, 8 females). Due to the ecological nature of the experiment, recordings of 3 children were interrupted by children's movements which introduced artefacts and in extent caused inconsistency among the experimental phases (e.g., they repeatedly touched their face with the hands thus affecting the temperature dynamics associated with the paradigm; or otherwise left the room searching for the parents). In addition, 2 children were excluded from the analyses because of unsuccessful engagement of the child with the toy resulting to the absence of the mishap. All children were accompanied by their mothers. They had normal cognitive development and no physical or psychological disease. Children were recruited from a database of parents who were contacted by paediatricians and covering middle socioeconomic and cultural urban areas in central Italy.

### Procedure

Every child participated in a modified version of the mishap paradigm, where children are led to believe to have broken a toy previously manipulated by the experimenter [3]. The experiment took place in a room which always had a stable temperature of 23°C, 50–60% humidity and no direct ventilation. Recordings took place across several months spanning from spring to winter. All children were dressed according to season and the majority of the thermal recordings took place in the afternoon from 4 to5 p.m., with the exception of one participant recording taking place at 10 a.m. Before the beginning of the testing period, the participants spent 10–20 min in the room for acclimatization purposes in order to allow their skin temperature to stabilize for baseline recordings [46]–[47]. It is important to note that the participants during the experimental period were seated on a chair with no body restriction. Prior to the experimental session, an adequate amount of time was provided as a ‘warm up’, where the experimenter played with the children using several toys, to psycho-emotionally habituate the child to the setting and the experimenter; This was performed initially in the presence of the experimenter and the parents. After this warm up period, the child was exposed to the modified “Mishap Paradigm” [3]. During this phase the experimenter placed the ‘warm up’ toys back in the box and introduced the manipulated toy. The robot was placed on a table in front of the child and the experimenter presented it as her favourite one and of particular personal value. During the experimental paradigm there were 5 distinct phases (1) “Baseline”: the experimenter engaged with the child using different types of interactive games (the warm up period), followed by the illustration of the ‘target toy’ (a robot chosen after a pilot study) along with several activities that could be performed by the toy; (2) “Playing”: the experimenter left the child to engage alone with the “target toy” prior to stating that it was of particular personal value (experimenter's favourite one); (3) “Mishap”: the child causes the robot to brake; (4) “Entrance Experimenter”: the experimenter re-entered the room while merely looking in silence at the broken toy for approximately 30 s and then asking: “What happened? Who did that? Did you break the toy?” (5) “Soothing”: the child was told cheerfully that the accident with the toy was not his/her fault since it was already broken and that the robot could be fixed (child and experimenter fix the toy together). These phases were carried out in all experimental sessions and were videotaped for later analyses. The entire session lasted an average of 20 minutes. During the experimental process the parents could observe the experiment behind a one way mirror.

### Materials and data acquisition

#### Toys and the mishap

During familiarization and baseline recordings a variety of toys were used such as plastic animals, a puzzle, a small doll house, a magic wand and a 3D book. The target toy used during the experimental run was a black and white robot of approximately 18 cm height. It had an ON/OFF button at the back that, when switched ON, made the robot walk and play music. In addition, the hands of the toy had a button which when pressed it caused the hand-grasping mechanism to open which was the “trigger” that was causing the mishap. To be more specific, the robot had some screws loose. The appearance of the toy did not provide any visual cues to the child that it was broken since it appeared exactly the same as a new one. When the child engaged with the robot and pressed a button on the arm, the hand-like grasping mechanism was disassembling rapidly.

#### Data acquisition

For the thermal data acquisition a digital FLIR SC3000 thermal infrared camera was used with temperature sensitivity of 0.02 K and a sampling rate of 0.02 s. To cancel noise effects caused by the sensor's shifts/oscillations the camera was blackbody calibrated and the camera was adjusted to record at 1 frame/s. For behavioral data acquisition two radio controlled cameras were used (Canon VC-C50iR) connected to two video-cassette recorders (BR-JVC). Then the two video signals were combined using a Pinnacle (liquid 6) system providing a two- or three split movies. Subsequently, with the use of the software “Interact Plus” (Mangold) behaviour of the child was encoded simultaneously with the ongoing movie. Finally, it is important to note that the results of 14 children are present in the table and not 15 since behavioral recordings did not take place for one particular child due to technical errors.

#### Behavioral data analysis

The children's distress was coded on a behavioral and verbal level starting from the initiation of each phase and finishing on the start of the following. The coding system for the child's responses was taken from studies of which the mishap design was practically identical to the current one [3]–[4], [10], [17]. Based on this scheme, the distress behaviour of the individual is coded according to five signs: avoiding gaze (child looked away, downward); facial expression (lip prolled in), repair, verbalisations (including negative self-evaluation and confession) and bodily tension (including bodily avoidance, hunched shoulders, head lowered, arms across body, covering face, fingers in mouth). For the first four signs the presence or the absence of the behaviour was considered whereas for bodily tension overall distress response was coded on a 3-point scale (0 = child shows no behavioral distress signs; 1 = child shows one sign of bodily tension; 2 = child shows two or more different signs of bodily tension). For the baseline phase, gaze avoidance, facial expression and bodily tension were only coded, for mishap, repair was also included and for the experimenters entrance phase, verbalizations were added. Moreover in the soothing phase we have interpreted as a distress sign the child's initial reaction to the fixed object (0 = child handles or touches the robot without reservation; 1 = child refuses to handle the fixed toy when prompted to do so). The rating for the children's distress was performed by two independent researchers with Kappa's alphas ranging from .72 to .80, *p*<.05. Finally, non-parametric, two related sample test was conducted in order to define if there was a significant difference in the behavioral signs of distress between baseline, mishap, the experimenter's entrance and soothing.

#### Thermal data analysis

Thermal video preprocessing and thermal data extraction was performed using the software ThermalCAM Researcher by FLIR (http://www.flir.com) and processed according to [17]. The final data sets were analyzed using the statistical software Statistical Package for the Social Sciences, version 17 (SPSS, Chicago, IL). Prior to any data analyses, the two operators divided the thermal video into 5 experimental phases and frames were extracted every 3–5 s. On average for each child at least 16 frames, equidistant in time, were used for “baseline”, 8 for “Playing”, 14 for “mishap”, 11 for the experimenter's entrance and 15 for the “soothing” phase. Side shots of the child or frames where the face was occluded were not used since trials showed that they induced serious temperature variations in the data. Only frontal shots or frames with constant angle of view along the whole recording were used. The Region of Interest (ROI) was selected as a circular area. The radius of the circle (0.5 cm) for the temperature extraction covered the nasal tip excluding the nostrils and was the same in all experimental frames for each individual child. This was essential, since ROI radius inconsistencies might induce significant thermal artifacts.

After the extraction of data points in each condition, Mann-Whitney U tests were performed (15/individuals×4condition contrast) in order to observe if there was a significant difference in the temperature between the five conditions. Furthermore, for each child the mean temperature of all data points in each condition was calculated (Baseline, Playing, Mishap, Entrance Exp., and Soothing). Subsequently, using the mean values of every condition for each child, group analysis was conducted using 1×5 one-way repeated measures ANOVA. In case ANOVA yielded a significant main effect, orthogonal difference contrasts were performed comparing each condition with the mean of the previous condition and using Bonferroni correction for multiple comparisons. Because of their orthogonality, these contrasts allowed to detect independently and specifically which conditions induced a significant change on the nasal tip temperature. Raw data for each individual can be retrieved from Dryad Digital Repository.

## Results

### Behavioral Results

Behavioral analyses showed signs of distress accompanied by feelings of guilt particularly during the mishap phase and the experimenter's entrance (Table 1). Non-parametric, two related sample test showed that children during baseline had a significant difference in distress levels compared to mishap (bodily tension, Wilcoxon test Z = 2.04, *p*<.05; avoiding gaze, McNemar test, χ^2^ (1) = 4.57 = *p*<.05; facial express χ^2^ (1) = 4.57 *p*<.05).

**Table 1:** Frequencies (percentage) for each variable coded and means and standard deviation for bodily tension in different phase: Baseline, Mishap, Entrance of the experimenter and Soothing (N = 14).

Variable	Baseline	Mishap	Entrance experimenter	Soothing
Avoiding gaze:	21.4	71.4	50.0	
Facial expression:	21.4	92.9	57.1	
Repair:		85.7	85.7	
Verbalizations:			42.9	
Relief to fixed object:				78.6
Bodily tension:	0.21 (0.43)	1.29 (0.83)	1.43 (0.85)	

Observing the experimenter's entrance phase, it seems that the level of distress does not change and children that confessed or judged themselves negatively showed more facial expressions of distress during the mishap phase (χ^2^ (1) = 10.28 *p*<.05) as well as a more reparatory behaviour when the experimenter entered (χ^2^ (1) = 7.14 *p*<.05). Finally, analyzing the soothing phase, it was observed that children expressed relief touching the fixed toy without reservation; only 3 children refused to handle the toy.

### Temperature Results

#### Individual Analyses

**Figure 1:**
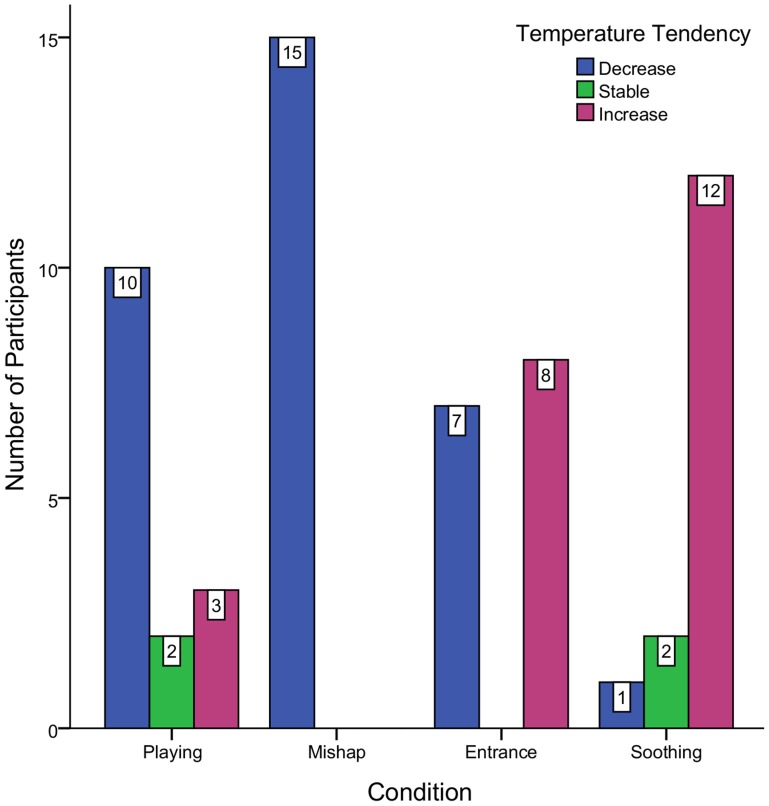
Clustered Bar Chart. Represents the temperature tendency for all children in each condition contrast for the Mann-Whitney U test.

**Figure 2:**
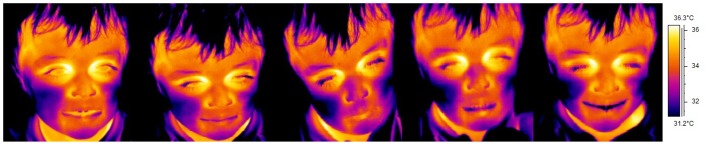
Five pictures of Child 7 showing the temperature change of the nasal tip. Baseline (1^st^ frame), playing (2^nd^ frame), mishap (3^rd^ frame), experimenter's entrance (4^th^ frame) and the soothing phase (5^th^ frame). Colour change from lighter to darker shades signify the temperature drop.

In order to examine at the individual level whether each experimental condition differed in temperature than the previous one, Mann-Whitney U tests were performed. Each experimental condition was compared with previous temperatures, resulting to 4 comparisons for all 15 individuals (Table 2). During playing the temperature of 8 out of 15 children significantly differed from baseline, with 6 having a temperature decrease and 2 an increase. The mishap phase resulted in 12 significant temperature drops whereas during the experimenter's entrance 4 out of 15 children reached a significant temperature decrease and 7 an increase. Finally during the soothing phase 12 children showed significantly higher temperatures than the previous conditions. Based on the Mann-Whitney U tests, Figure 1 represents a summary plot of temperature direction for each child. Furthermore having in mind that measurements were made in different seasonal times and since interest is given on the change of temperature across conditions; for purposes of illustration the evolution of temperature as a function of time for each child was plotted on a scale with a range of 7 degrees and 0.5 degrees of major increment (see also Figure S1). Figure 2 shows the variations of the facial temperature in a representative child (#7).

**Table 2:** Mann-Whitney U tests, for all 15 individuals compared to preceding conditions (* = p<.05).

Child	Condition	*U*	*P*	*Z*	*r*	*Md*	*n*	*Vs.Md*	*n*
1	Play Vs.	9	.002	−3.03	−.63*	31.3	6	31.5	17
	Mishap Vs.	18	.001	−3.12	−.56*	31.1	7	31.5	23
	Entrance Vs.	31	.00	−3.50	−.56*	31.1	9	31.4	30
	Soothing Vs.	232	.08	−1.77	+.24*	31.7	17	31.3	39
2	Play Vs.	45	.71	−.391	±.09	34.9	9	34.9	11
	Mishap Vs.	34	.00	−3.42	−.60*	34.5	12	34.9	20
	Entrance Vs.	64	.03	−2.19	+.35*	35.05	8	34.8	32
	Soothing Vs.	8	.00	−5.25	+.72*	35.5	13	34.9	40
3	Play Vs.	0	.00	−3.3	−.73*	29.5	5	30.8	15
	Mishap Vs.	116	.10	−1.63	−.26	29.6	17	30.5	20
	Entrance Vs.	1	.00	−3.86	+.58*	32.95	6	30.3	37
	Soothing Vs.	3	.00	−5.67	+.74*	33.9	15	30.5	43
4	Play Vs.	33	.00	−5.16	+.73*	33.6	33	33.1	17
	Mishap Vs.	185	.04	−2.06	−.26*	33.3	12	33.6	50
	Entrance Vs.	218	.00	−2.92	−.33*	33.2	14	33.6	62
	Soothing Vs.	392	.74	−.33	±.04	33.5	11	33.5	76
5	Play Vs.	2	.00	−.38	−.73*	34.7	7	35.1	20
	Mishap Vs.	81	.00	−3.78	−.56*	34.6	18	35.1	27
	Entrance Vs.	224	.98	−.02	+.00	34.95	10	34.8	45
	Soothing Vs.	213	.06	−1.92	+.23*	35.0	12	34.8	55
6	Play Vs.	10	.27	−1.28	±.37	32.8	5	32.8	7
	Mishap Vs.	78	.74	−.34	−.07	32.7	14	32.8	12
	Entrance Vs.	51	.00	−4.08	+.63*	33.4	16	32.8	26
	Soothing Vs.	108	.00	−3.76	−.50*	32.5	15	32.9	42
7	Play Vs.	8	.02	−2.53	−.61*	34.6	5	34.7	12
	Mishap Vs.	1	.00	−5.02	−.85*	33.6	17	34.7	17
	Entrance Vs.	213	.22	−1.22	−.17	34.1	16	34.3	34
	Soothing Vs.	144	.00	−3.62	+.45*	34.6	15	34.2	50
8	Play Vs.	43	.10	−1.69	−.34	32.75	12	33.0	12
	Mishap Vs.	35	.00	−4.76	−.73*	32.3	19	32.9	24
	Entrance Vs.	282	.73	−.35	+.05	32.5	14	32.4	43
	Soothing Vs.	116	.00	−5.46	+.62*	33.1	21	32.4	57
9	Play Vs.	20	.02	−2.40	−.48*	35.8	6	36.0	19
	Mishap Vs.	6	.00	−4.73	−.78*	35.7	12	35.9	25
	Entrance Vs.	66	.00	−3.68	−.53*	35.6	12	35.8	37
	Soothing Vs.	95	.00	−3.05	+.39*	36.1	10	35.7	49
10	Play Vs.	20	.39	−.93	+.24	33.25	6	33.2	9
	Mishap Vs.	75	.13	−1.57	−.28	33.1	15	33.2	15
	Entrance Vs.	10.5	.00	−4.4	−.69*	32.75	10	33.2	30
	Soothing Vs.	161	.36	−.95	+.13	33.2	10	33.1	40
11	Play Vs.	9	.00	−2.86	−.57	33.3	5	33.55	20
	Mishap Vs.	00	.00	−5.16	−.83*	32.0	14	33.4	25
	Entrance Vs.	55	.00	−2.88	+.42*	33.8	8	33.3	39
	Soothing Vs.	145	.00	−3.67	+.46*	33.8	16	33.3	47
12	Play Vs.	4.5	.00	−2.89	+.66*	33.3	6	32.9	11
	Mishap Vs.	5.5	.00	−3.85	−.75*	32.2	9	33.1	17
	Entrance Vs.	11	.00	−5.71	−.82*	31.9	22	32.9	26
	Soothing Vs.	62	.00	−4.18	+.54*	33.7	12	32.1	48
13	Play Vs.	6	.00	−3.42	−.66*	32.3	6	32.5	21
	Mishap Vs.	11	.00	−4.80	−.76*	32.1	13	32.4	27
	Entrance Vs.	68	.26	−1.27	−.17	32.3	5	32.35	40
	Soothing Vs.	172	.47	−.716	±.1	32.3	9	32.3	45
14	Play Vs.	93	.93	−.124	−.02	33.85	6	33.9	32
	Mishap Vs.	244	.95	−.07	−.00	33.8	13	33.9	38
	Entrance Vs.	103	.00	−3.1	+.39*	33.95	10	33.9	51
	Soothing Vs.	369	.00	−5.72	+.57*	34	37	33.9	61
15	Play Vs.	70	.42	−.915	−.16	28.05	8	28.1	22
	Mishap Vs.	6.5	.00	−5.41	−.81*	27.8	15	28.1	30
	Entrance Vs.	83.5	.00	−3.46	+.46*	28.2	11	28.0	45
	Soothing Vs.	94.5	.00	−5.05	+.59*	28.4	17	28.0	56

#### Group Analyses

**Figure 3:**
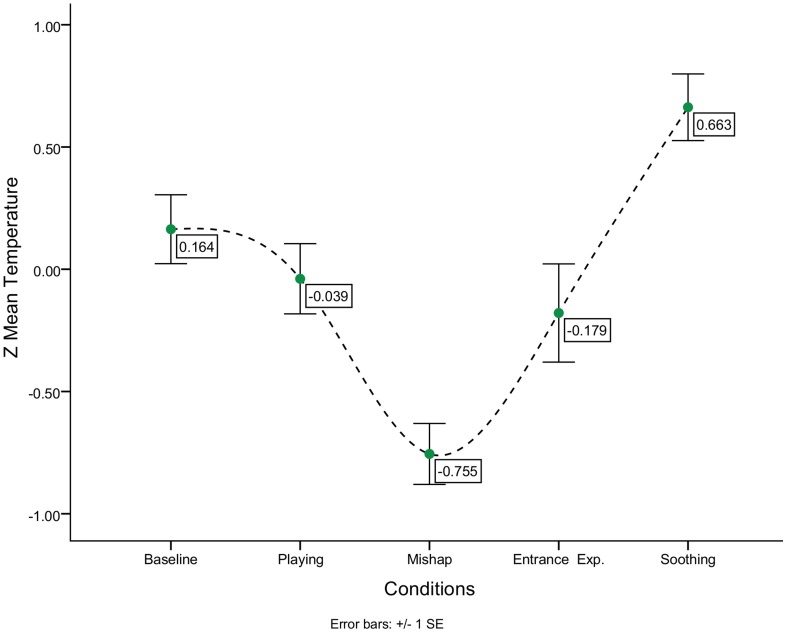
Group graph: Z Mean temperature for each condition.

One-way repeated measures ANOVA was conducted in order to examine at the group level if there was a significant difference in the mean scores of the 15 children between the 5 conditions. Results showed that there was a significant main effect for condition [Wilks' Lambda = .404, *F* (4, 11) = 4.049, *p*<.0005, multivariate partial eta squared = .596]. Within conditions contrasts yielded a significant nasal tip temperature decrease for mishap [*F* (1, 14) = 14.178, *p*<.0005, multivariate partial eta squared = .503, (*M* = 32.63, *SD* = 1.93)], (Table 3, 4/Figure 3). The mean difference between the mishap phase and baseline was 0.44°C and between the mishap phase and the playing phase was 0.27°C. Playing and the Experimenters Entrance (M = 33.03, *SD* = 1.79) did not differ significantly from the previous conditions (*p*>.05). On the contrary, the soothing phase (*M* = 33.4102, *SD* = 1.83) differed significantly from previous conditions [F (1, 14) = 6.484 *p*<.0005, multivariate partial eta squared = .317]. Average temperature during the soothing phase differed 0.3°C from baseline, 0.5°C from playing, 0.8°C from mishap, and 0.4°C from experimenters entrance. Post-hoc power analyses was performed using the G*Power software as suggested by [59] to examine whether the conducted experiment had enough statistical power to detect an effect for condition [60]. With sample size of 15 and an eta squared of .596 the study had enough power [61] to detect an effect for experimental condition (*d* = .81).

**Table 3:** Descriptive statistics for the mean temperature of each condition.

	Mean	Std. Deviation	N
Baseline	33.08	1.95	15
Playing	32.91	2.02	15
Mishap	32.64	1.93	15
Entrance Exp.	33.03	1.78	15
Soothing	33.41	1.83	15

**Table 4:** Repeated measures ANOVA for within conditions contrasts for 15 children (* = p<.05; ** = p<.005) a computed using alpha = .05.

Source	Factor 1	Type III Sum of Squares	df	Mean Square	F	Sig.	Partial Eta Squared	Observed Power^a^
Condition	Playing vs. Baseline	.401	1	.401	2.787	.117	.166	.342
	Mishap vs. Previous	1.927	1	1.927	14.178	.002**	.503	.938
	Entrance Exp. vs. Previous	.368	1	.368	.526	.480	.036	.104
	Soothing vs. Previous	3.655	1	3.655	6.484	.023*	.317	.659
Error (condition)	Playing vs. Baseline	2.016	14	.144				
	Mishap vs. Previous	1.903	14	.136				
	Entrance Exp. vs. Previous	9.786	14	.699				
	Soothing vs. Previous	7.892	14	.564				

## Discussion

The purpose of the present study was to characterize the autonomic response associated with guilt in early childhood. Since, from a functional perspective, guilt-relevant behaviours are exhibited by young children under the appropriate situations the mishap paradigm was used for eliciting such kind of behaviours. Moreover, in order to preserve the children's naturalistic and spontaneous behaviour, physiological assessments were made by fIRI, a non invasive method, capable of detecting the skin temperature variations expected to occur in subjects involved in emotional contexts. Unlike other studies of ANS arousal in emotion, physiological recordings in the current study were made with fIRI on a continuous physiological line, supported by observations of behavioral nature. This study provides additional evidence for the reliability of the nasal region in observing affective signs and may provide ground for the physiological study of guilt in adults.

Current behavioral findings suggest that all children involved in the paradigm experienced emotional distress. According to literature [3], [4], [10], [17], [50] the critical guilt-relevant pattern of behaviors, including expressive signals and reparative actions, were shown by all the children in the appropriate phases of the task, such as after transgression and at the entrance of the experimenter. Physiological data reflected that experience. It was observed that at the group level the temperature of the nose during mishap significantly differed from the previous conditions and particularly in contrast to baseline, dropping an average of 0.44°C. On the contrary, temperature during playing and the experimenter's entrance did not provide statistical significance in comparison to the previous conditions temperatures. In addition, soothing also evoked a significant difference from previous conditions with the highest temperature difference observed, 0.8°C compared with mishap. So, the paradigm confirmed to be capable of eliciting the guilt experience in early childhood. The above results are in accordance with the hypotheses.

Avoiding gaze, facial expression and bodily tension, after the transgression occurred, were shown by the majority of the children and were associated with the temperature drop of the nasal tip. We could suppose that mishap situations are experienced by the child as threatening and thus trigger the defensive physiological component of the autonomic nervous system, the ‘fight or flight’ response controlled by sympathetic nervous system [33], [49], [51]. This physiological phenomenon of temperature drop was shown by previous studies not to be related to heavy breathing [46] or movement artifacts [47] but it has been suggested [30], [46] to be related to the presence of arteriovenous anastomoses [50] in the nasal region, constricted by efferent sympathetic nerves. Arteriovenous anastomoses have been argued to be even more present in the skin of fingers [50] and by using fIRI it has been observed that negative emotional states induced a marked decrease in participants' fingers of up to 2°C [52]. During threat or psychological stress, the SNS causes certain physiological changes to take place through the release of epinephrine in the blood stream and under this occasion subcutaneous blood vessels constrict, leading to external heat from the surface of the skin to drop [39]]–[[42]. Emotional or thermoregulatory sweating could not have occurred on the nasal region since no perspiration spots were detected during temperature analyses of the region of interest [47]. However it is hard to completely rule out the possibility that temperature changes were a product of altered blood flow, dependent on systemic arterial blood pressure and vascular resistance or conductance in the bed [30], since – to the best of our knowledge- no work exists on arterial pressure in children during similar experimental paradigms.

Sympathetic activity can still be inferred during the experimenter's entrance despite the fact that the temperature of the nose started rising having only a slightly lower temperature from baseline of 0.05°C. This could be accounted to the fact that during this phase, where children were facing the adult, they were asked about the cause of damage and typically tried to repair the wrongdoing by apologizing or confessing. This is probably why a lower temperature than baseline was still observed, backed up also by the behavioral observations.

Lastly, it is important to note that during soothing the average temperature was 0.3°C higher than baseline and 0.8°C higher than mishap. According to a sensible interpretation, the child's distress was soon neutralized, or even overcompensated, during the soothing condition, which entailed social interaction and mishap restoration.

**Figure 4:**
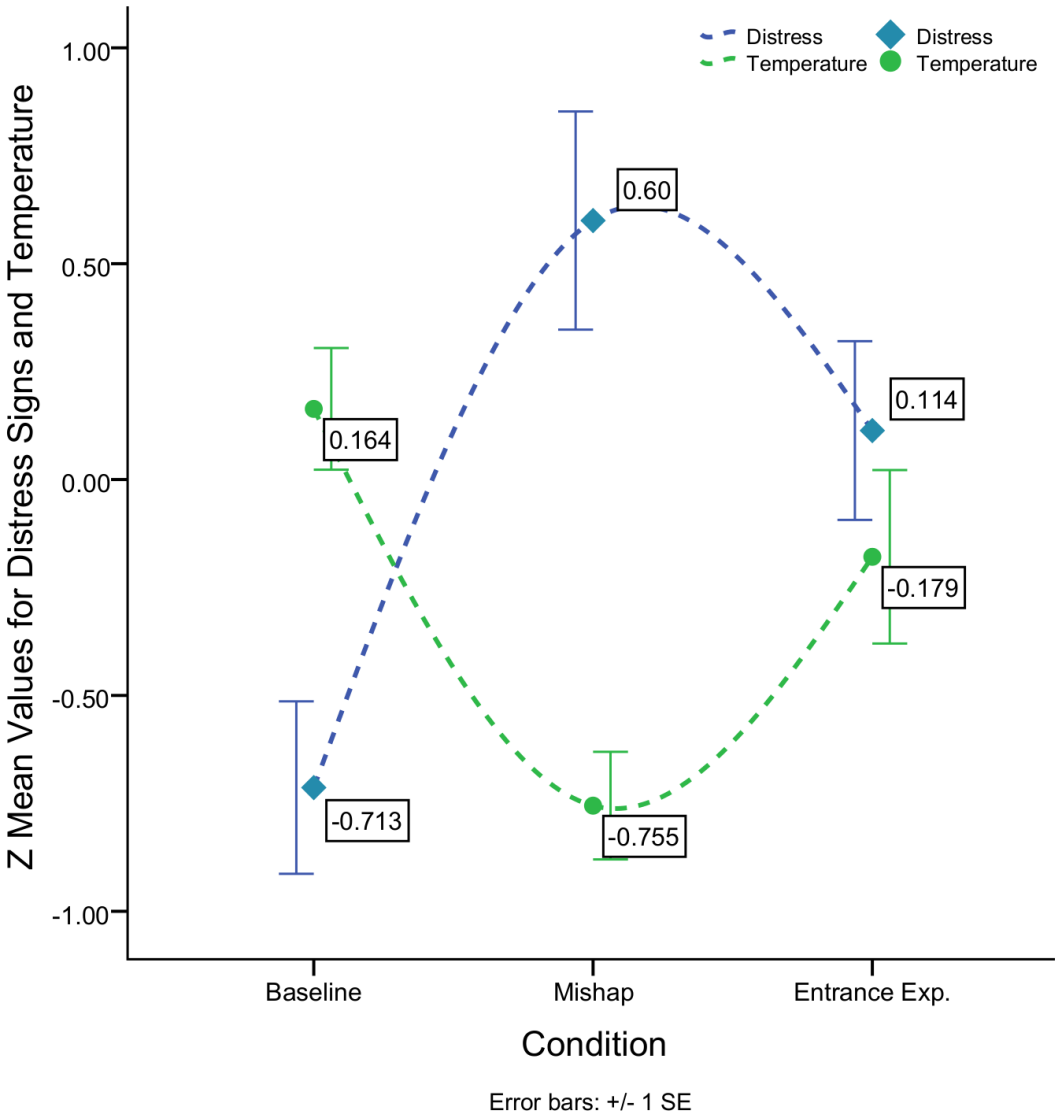
Group graph: Z mean behavioral distress signs and Temperature change for Baseline, Mishap and Experimenter's entrance.

To summarize, behavioral results obtained by the study confirms the evidence of children's distress after the transgression, an observation supported by a great amount of research signalling early guilt. Physiological data showed an inverse relationship with the behavioral evidence with the temperature of the nasal tip dropping during mishap while levels of distress rose and returning to baseline temperatures after distress was reduced (Figure 4). This finding is consistent with a number of studies reporting significant nasal temperature drops due to unpleasant events inferring sympathetic activity (however, see [30] for seemingly opposite results in infants). For example [45], reported a temperature drop of 0.5°C as a consequence of a loud noise [46], recorded a temperature decrease of 0.2±0.1°C by exposing monkeys to a threatening person, and finally [43], reported a nasal tip temperature drop of 0.32–0.56°C due to increased mental workload. Moreover, the temperature increase during soothing is also in line with the study by [44], in which they reported a temperature increase of the nose during social interaction of 0.1°C.

All together, these results showed evidence of a coherent pattern of physiological variations underlying guilt-relevant behaviors. The findings allow to claim that young children experience distressful feeling when involved in the mishaps paradigm, providing some support to the functionalistic view of early moral development.

It would be important to note that no behavioral coding was performed for the playing condition, since the scope of the study regarded the autonomic print of the transgression, the adult's entrance, as well as the restoration of the wrongdoing during soothing. Although the playing phase did not reach statistical significance compared to baseline, a slight temperature decrease was observed despite the initial expectation. During play the child might have experienced a state of amusement and happiness both previously characterised by alpha-adrenergic increase [62].

In addition, observations between behavior and temperature were not based on correlation analyses but on a separate examination of the quantitative analyses of the two measures. Although physiology arises in occasions of socio-emotional or physical danger, the reason of its existence is not to cause a particular behaviour but rather to support it. Behavioral engagement and the multiple strategies followed by the individual are relevant to the cognitive context of the situation [63]–[64] whereas on the contrary, only two subdivisions of the ANS apply in a variety of environmental settings [35], [65]. In line with this argument, is the experiment by [64]. Whereas individuals received epinephrine (adrenaline) shots and were exposed to euphoric and angry situations they addressed not only two different emotions but also they exhibited two different behaviors. Whereas autonomic arousal can be similar in different situation behavior hardly ever stays constant and varies largely based on the environmental requirements. This is the reason why behavior is coded on a plethora of observations (e.g. bodily, verbal, facial) whereas physiology, only, on the competing subdivision of the ANS. In extent very few emotions can be specified by their physiological mark. However if multiple physiological measures (e.g. breathing, heart rate, GSR) are taken into account [17] along with the speed of onset, intensity and duration then it might be possible that more emotions can be defined [66].

Finally, fIRI proved to be a versatile and sensitive physiological tool, extending the already existing literature of moral emotions from developmental studies to investigations of the autonomic nature of emotions. The availability of physiological measures by fIRI helps to consolidate behavioral assessments which have been so far, the only informative channel for studying moral development and self conscious emotions. In extent is especially useful in cases where behavioral strategies are prevented, such as among paralyzed patients, where individuals can still feel emotions as prior to their injury, but can no longer show behavioral responses in the same manner [53]. In contrast to paralysis, a condition called pure autonomic failure in which the nervous system no longer regulates the heart or the internal organs, results in patients' reports of diminished emotional experiences [54]. Although these patients have no difficulty articulating what emotion is felt [55]. ANS physiological responses seem to be more closely linked to emotional experience than to behavioral engagement; for this reason ANS measures should be essential tools in emotion research [18], [21], [22]. The simplicity of using fIRI for observing the psycho-physiological correlates of emotional experience warrants its use in developmental, comparative and evolutionary studies, particularly in the non-invasive and ecologically valid study of child behaviour [56], [57]. More to the point of this study, its sensitivity to autonomic responses associated with moral experience could be particularly valuable when investigating children who have an impaired ability to exhibit some range of social-cognitive and empathic skills, such as those affected by Autism Spectrum Disorders (ASD). Moreover, fIRI might even be a valuable tool in clinical research in the early diagnosis of psychopathy since these individuals have difficulty in learning morally normative responses due to impairments in emotional aversive learning [20]. In extent, although psychopaths can show complex emotions such as embarrassment, they face great difficulties in comprehending situations imposing guilt [58]. As in many cases of pathology, though, the earlier such conditions are diagnosed, the greater is the ability to offer useful prognoses in the form of behavioral interventions and counseling.

## Conclusions

Emotions have a strong physiological mark which accompanies the feeling experience. From the analyses it was observed that complex emotions such as guilt are characterized by a temperature drop, compared to baseline measures. This incidence of nasal tip thermal reduction implies sympathetic arousal through peripheral vasoconstriction related to the experience of guilt, whereas the temperature restoration during soothing could be due to parasympathetic activity and vascular restoration. Physiological states are the supporting mechanisms for the behavioral component of moral emotions. Thus, the integration of physiological elements with behavioral grounding should be crucial in the observation and interpretation of affective emotional states, as those involving the formation of the moral self, the maturation of the neo-cortex and the healthy function of the sub-cortex.

## Supporting Information

Figure S1 **Line Graphs.** Graphs representing the evolution of temperature over time for each child. The coloured vertical lines represent the onset of each condition (purple = playing, red = mishap, green = entrance experimenter, blue = soothing).
(TIF)
