# Supplementary figures and images for: The Autonomic Signature of Guilt in Children: A Thermal Infrared Imaging Study
Source: PLoS One. 2013 Nov 19;8(11):e79440. doi: 10.1371/journal.pone.0079440 (PMC3834185; doi:10.1371/journal.pone.0079440)

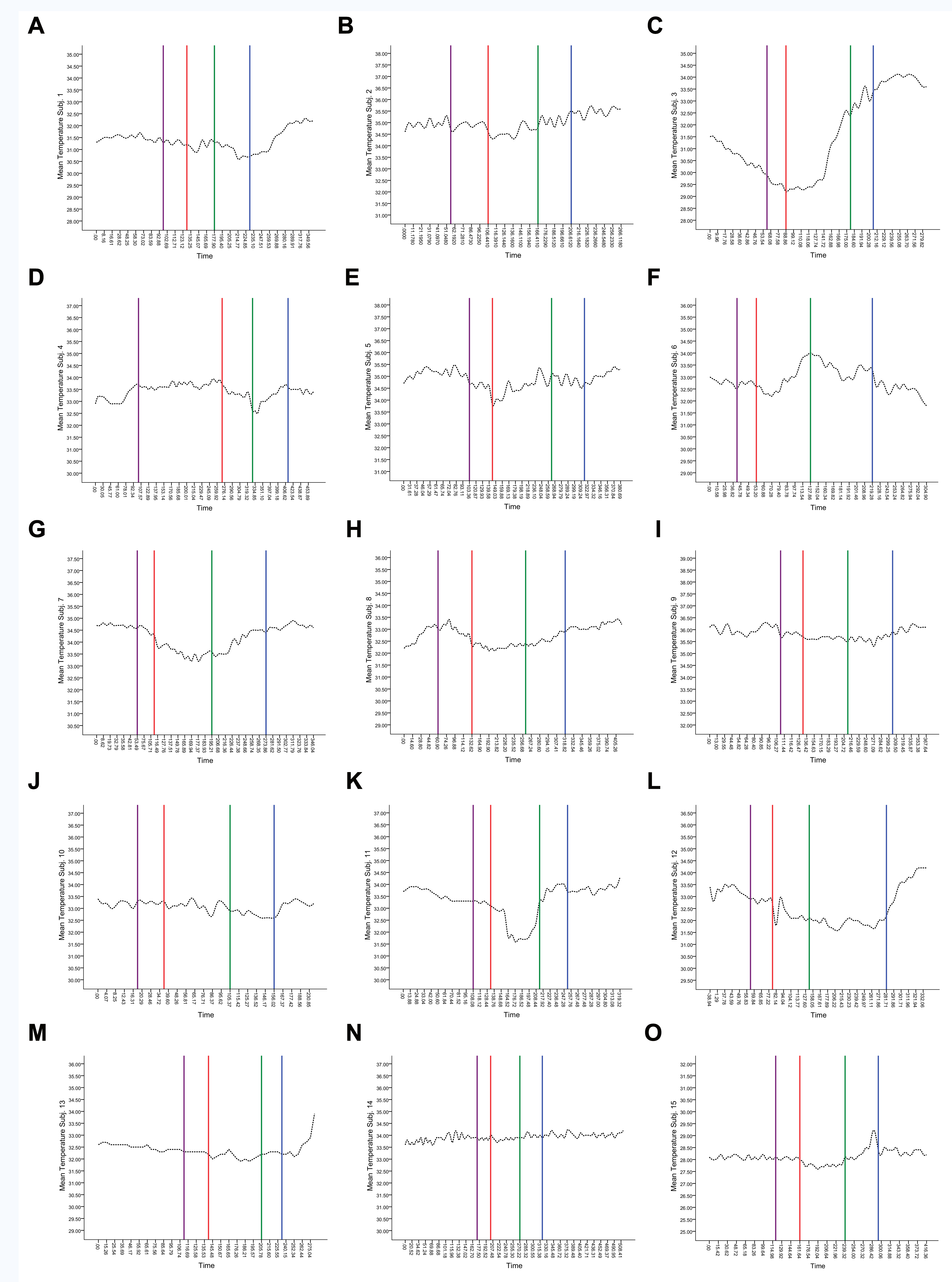

Supplement: Figure S1 — Line Graphs. Graphs representing the evolution of temperature over time for each child. The coloured vertical lines represent the onset of each condition (purple = playing, red = mishap, green = entrance experimenter, blue = soothing). (TIF) [file pone.0079440.s001.tif]
